# P-323. Evaluation of WHO Five Moments Compliance Among Nursing, Paramedical Staff, and Emergency Medicine Technicians in a Tertiary Care Hospital in India: Expected vs. Actual

**DOI:** 10.1093/ofid/ofae631.526

**Published:** 2025-01-29

**Authors:** Alvin Sunny, Jose J Kochuparambil

**Affiliations:** Mary Queen's Mission Hospital, Kottayam, Kerala, India; MQMH, Pala, Kerala, India

## Abstract

**Background:**

In tertiary care hospitals of a resource limited country like India, understanding hand hygiene (HH) compliance among nursing, paramedical staff, and emergency medical technicians according to the WHO Five Moments (5M) protocol is critical for improving infection control practices and patient outcomes.

**Methods:**

Utilizing a pre-designed structured hand hygiene audit tool, this study conducted over 300 hours of direct HH observations between 2022 and 2022 at a tertiary care hospital in India. A total of 150 nursing staff, 60 paramedics, and 20 emergency medical technicians were included in the study cohort. Compliance with WHO 5M guidelines, including hand washing (50 seconds) and alcohol rub (25 seconds), was meticulously observed and recorded. Following the initial observation period, a targeted training program on the WHO Five Moments protocol was implemented for all healthcare professionals.

**Results:**

A notable discrepancy between expected and actual compliance with HH protocols was evident among the observed healthcare professionals. Compliance varied across the groups: Among the 150 nursing staff, compliance rates were 55%; among the 60 paramedics, compliance rates were 40%; and among the 20 emergency medical technicians, compliance rates were 35%. Despite the established duration for HH procedures, all groups demonstrated suboptimal adherence to HH guidelines. Adherence to expected compliance increased the overall time per patient encounter by 5.5-11 minutes, highlighting the need for improved HH practices. Subsequent observations conducted six months after the training revealed a significant improvement in HH compliance rates across all groups: nursing staff demonstrated an increase to 81.3% paramedics to 68.3%, EMTs to 58.5%.

**Conclusion:**

The study emphasizes the urgent need to address the gap between expected and actual HH compliance among healthcare professionals in Indian tertiary care hospitals. Enhanced educational interventions and reinforcement of HH practices are imperative to minimize the risk of healthcare-associated infections and improve patient safety outcomes.

**Disclosures:**

**All Authors**: No reported disclosures

